# The Duality of Cdk5: A Master Regulator in Neurodevelopment and a Hijacked Oncogene in Cancer

**DOI:** 10.3390/cells14231876

**Published:** 2025-11-27

**Authors:** Yoshiaki V. Nishimura, Takeshi Kawauchi

**Affiliations:** 1 Division of Neuroscience, Faculty of Medicine, Tohoku Medical and Pharmaceutical University, 1-15-1 Fukumuro, Miyagino-ku, Sendai, Miyagi 983-8536, Japan; yvnishimura@tohoku-mpu.ac.jp; 2 Department of Adaptive and Maladaptive Responses in Health and Disease, Graduate School of Medicine, Kyoto University, Medical Innovation Center (3F), 53 Kawahara-cho, Shogoin, Sakyo-ku, Kyoto 606-8507, Japan; 3 Department of Physiology, Keio University School of Medicine, 35 Shinanomachi, Shinjuku-ku, Tokyo 160-8582, Japan

**Keywords:** Cdk5 (cyclin-dependent kinase 5), neurodevelopment, cancer, neuronal migration, context-dependent kinase

## Abstract

**Highlights:**

**What are the main findings?**

- Cyclin-dependent kinase 5 (Cdk5) exhibits a functional duality: it is an essential regulator of neurodevelopment, but it is frequently repurposed as an oncogene in cancer to drive proliferation, metastasis, and therapeutic resistance.
- The functional output of Cdk5 is context-dependent, determined by its upstream regulation, choice of activator, and subcellular localization. Notably, it can act as a nuclear tumor suppressor in gastric cancer, in contrast to its cytoplasmic oncogenic roles.

**What are the implications of the main findings?**

- The duality of Cdk5 presents an opportunity and a significant challenge for cancer therapy. Novel strategies are needed to distinguish its oncogenic activities from its essential physiological functions, ensuring both efficacy and safety.
- By bridging neurodevelopment and cancer biology, this cross disciplinary synthesis offers a unified framework that clarifies shared mechanisms and supports reciprocal progress in both fields.

**Abstract:**

Cyclin-dependent kinase 5 (Cdk5) is an atypical serine/threonine kinase distinct from classical cell cycle regulators. Its activity is highest in the nervous system and essential for development, but its functions in other tissues, particularly in cancer, are increasingly being elucidated. This review explores the functional duality of Cdk5 by comparing its constructive role in neurodevelopment with its repurposed oncogenic function in cancer. In neurodevelopment, Cdk5 orchestrates nearly every stage of brain construction, including neuronal differentiation, migration, and synaptic plasticity. However, in many cancers, this neurodevelopmental toolkit is repurposed, and aberrantly activated Cdk5 promotes proliferation, metastasis, and therapeutic resistance in diverse solid tumors. Cdk5 also actively shapes the tumor microenvironment by promoting angiogenesis and modulating immunity. Notably, this oncogenic function is not universal, as Cdk5 exhibits its duality even within the context of cancer; it acts as a tumor suppressor in gastric cancer upon nuclear localization. Taken together, these lines of evidence underscore that Cdk5 is a context-dependent kinase whose output is determined by upstream regulation, subcellular localization, and the cellular environment. This review discusses the molecular basis of its dual role and highlights both the potential and complexity of Cdk5 as a therapeutic target in oncology.

## 1. Introduction: The Discovery and Characteristics of the Atypical Cyclin-Dependent Kinase Cdk5

Cyclin-dependent kinase 5 (Cdk5) is a proline-directed serine/threonine kinase identified in 1992 [1,2,3,4]. Due to its high amino acid sequence homology (approximately 60%) with Cdk1 (Cdc2), it was initially presumed to be involved in the cell cycle regulation. However, subsequent research revealed that Cdk5 is an “atypical” kinase with properties and functions that clearly distinguish it from other Cdk family members. Whereas classical Cdk family are activated by binding to regulatory subunits known as cyclins, primarily in proliferating cells to control cell cycle progression, Cdk5 exhibits its highest expression levels and kinase activity in differentiating and mature post-mitotic neurons of the central nervous system [5].

**Figure 1:**
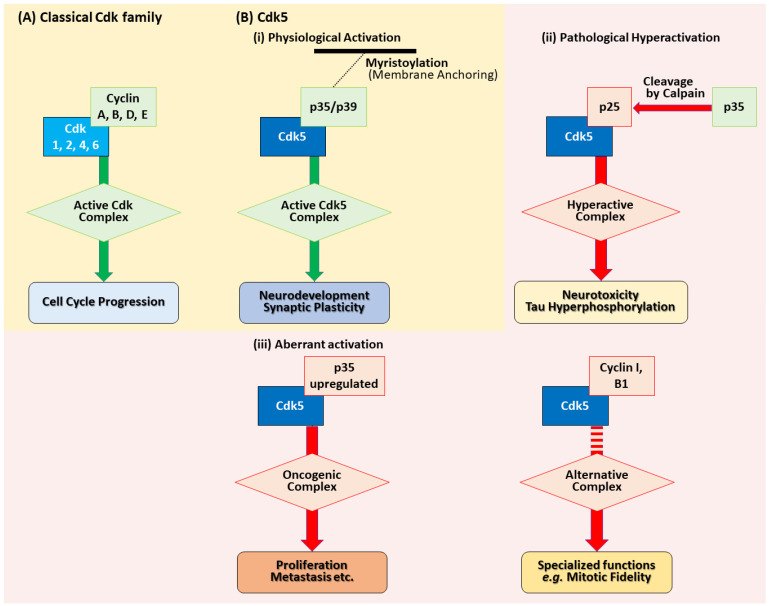
Distinct activation mechanisms of classical Cdk family and the atypical kinase Cdk5. (**A**) Classical Cdk family are activated by cyclins in proliferating cells to drive cell cycle progression. (**B**) Cdk5 activation is context-dependent and mediated by non-cyclin activators. (i) In neurons, Cdk5 binds its physiological activators, p35 (encoded by CDK5R1) and p39 (encoded by CDK5R2), directing its function in neurodevelopment. (ii) In neurodegeneration, calpain-mediated cleavage of p35 to the stable p25 fragment leads to a hyperactive, mislocalized Cdk5/p25 complex that drives neurotoxicity. (iii) In cancer, Cdk5 is aberrantly activated, primarily through p35 overexpression, to promote tumorigenesis. It can also partner with alternative cyclins (e.g., Cyclin I, B1) for specialized functions.

This neuronal specificity arises from Cdk5’s unique activation mechanism (Figure 1). Unlike canonical Cdk family, Cdk5 does not typically partner with classical cell cycle-associated cyclins, such as cyclins A, D, and E, under physiological conditions in neurons. Instead, it is predominantly activated by the neuron-specific non-cyclin activators, p35 (encoded by CDK5R1) and p39 (encoded by CDK5R2) [6,7,8,9,10]. While p35 and p39 are highly homologous and have functionally overlapping roles [9,10,11], their spatiotemporal expression patterns within the brain are distinct. p35 is broadly expressed at high levels during embryonic development across brain regions, with the strongest expression in the cerebral cortex and declining postnatally. By contrast, p39 shows region-dependent dynamics: it is high embryonically in the cerebellum, brainstem, and spinal cord but low in the embryonic cortex, where it increases postnatally and peaks in early postnatal stages (P7–P14) [12]. These spatiotemporal differences suggest that p35 and p39 play distinct or cooperative roles depending on the developmental stage and brain region.

The stability and localization of these activators are tightly regulated, and their dysregulation is associated with pathological conditions. For instance, in Alzheimer’s disease, amyloid-β can induce S-nitrosylation of p39, leading to its degradation and contributing to synaptic dysfunction [13]. Furthermore, p35 and p39 are anchored to the cell membrane via myristoylation of a glycine residue at their N-terminus [14]. This tethers the activated Cdk5 complex to the membrane, allowing it to efficiently participate in membrane-associated intracellular signaling and cytoskeletal regulation [15]. Consistent with this, single-cell transcriptomic analyses of adult tissues have shown that while Cdk5 transcripts are detectable across multiple non-neuronal cell types, its canonical activators, p35 and p39, remain largely neuron-enriched [16].

Indeed, the partner choice of Cdk5 is context-dependent. In non-neuronal cells, Cdk5 can also associate with a noncanonical cyclin, Cyclin I (CCNI), for example, in kidney podocytes, and possibly with Cyclin I-like (CCNI2), which affects its subcellular localization and promotes survival-related signaling [17,18,19,20]. Moreover, in proliferative settings, including cancer, emerging evidence indicates that Cdk5 can form a rare complex with cyclin B1, playing a role in the regulation of mitotic fidelity (see Section 4) [21].

Given its reliance on neuron-enriched activators and predominant expression in the nervous system, Cdk5 has extensively been studied as a key kinase involved in neuronal differentiation and the maintenance of mature neuronal functions, rather than in cell proliferation. However, recent studies have shown that the roles of Cdk5 are not limited to the nervous system. Particularly in the pathological state of cancer, its regulation is disrupted, and it plays a central role in malignant transformation and tumor progression. This review will first explore the integrative roles of Cdk5 in normal neurodevelopment and then examine how these functions are repurposed in cancer to drive disease progression. This will highlight the functional diversity and context-dependency of this single molecule, and we will discuss its clinical significance and potential as a therapeutic target. This comparative analysis is intended to bridge the conceptual gap between Cdk5’s functions in neurodevelopment and tumorigenesis, offering insights into the molecular basis of its functional duality. In the next section, we outline the multi-layered upstream system that governs Cdk5 activity, establishing the basis for its context-dependent outputs.

## 2. Upstream Regulation of Cdk5 Activity

The functional versatility of Cdk5, acting as both a key kinase in the construction of the nervous system and a potent driver of oncogenesis, relies on a sophisticated, multi-layered regulatory system that modulates its activity in a spatiotemporally specific manner. While the binding of its activators, p35 and p39, is the primary switch for kinase activation, this event is itself governed by upstream signals that control the expression, stability, localization, and post-translational modification of the core components of the active Cdk5 complex. This chapter outlines these key regulatory layers. We first cover transcriptional control (Section 2.1), then post-translational regulation of activators (Section 2.2) and Cdk5 itself (Section 2.3), and finally dedicated negative regulators (Section 2.4).

### 2.1. Transcriptional Control of Activator Expression

The expression of Cdk5 activators is highly context-dependent. A striking example is observed in medullary thyroid carcinoma (MTC), a neuroendocrine tumor originating from the calcitonin-secreting C cells (parafollicular cells) of the thyroid. In human MTC, oncogenic signaling, frequently driven by gain-of-function mutations in the RET receptor tyrosine kinase, leads to the overexpression of the Cdk5 activator p35 and subsequent Cdk5 hyperactivation. Crucially, mimicking this state in a conditional mouse model—through C cell-specific overexpression of p25, the hyperstable fragment of p35—was sufficient to induce lethal MTC. Together, these findings highlight that an increase in the activator’s abundance, whether through transcriptional upregulation or enhanced stability, is a key oncogenic event [22].

### 2.2. Post-Translational Regulation of Activator Stability and Function

Beyond transcriptional programs, activator abundance and function are acutely tuned by post-translational mechanisms. A critical feature of Cdk5 regulation is the intrinsic instability of its activators. p35 is a short-lived protein with a half-life of approximately 20–30 min, primarily degraded via the ubiquitin–proteasome pathway. Intriguingly, this degradation forms part of an autoregulatory loop: inhibition of Cdk5 or mutation of its phosphorylation sites on p35 stabilizes the activator, indicating that Cdk5 activity promotes its own termination by triggering p35 turnover [23]. Beyond phosphorylation-mediated degradation, p35 function is also modulated by other post-translational modifications. For example, SUMOylation at Lys246 and Lys290 by SUMO2 enhances the activity of the p35/Cdk5 complex. This modification is induced by oxidative stress, providing a mechanism for the rapid tuning of Cdk5 output in response to environmental cues [24]. In addition, S-nitrosylation of p35 at Cys92 has been reported to accelerate its proteasomal degradation, thereby providing a nitrosative-stress–sensitive brake on Cdk5 activity [25]. Beyond stability, p35 localization is also dynamically regulated. It can be actively imported into the nucleus by importin family proteins, a process that can involve its dissociation from Cdk5, suggesting an independent nuclear role for p35 beyond Cdk5 activation [26].

### 2.3. Post-Translational Modifications of Cdk5

In parallel, Cdk5 itself is post-translationally modified (some sites remain debated) providing additional control over catalytic output. These include acetylation (e.g., at Lys33) and S-nitrosylation (e.g., at Cys83), both of which have been shown to directly impact its kinase activity [27,28]. However, the most extensively studied—and debated—modification is phosphorylation.

One such site is Tyr15, located within the ATP-binding pocket. It was proposed that Sema3A signaling recruits the Src-family kinase Fyn to phosphorylate Cdk5 on this residue, thereby enhancing its kinase activity [29]. However, a later study demonstrated that Tyr15 phosphorylation occurs only in the monomeric, inactive form of Cdk5 and is inhibited by the activator binding, suggesting an alternative role for Fyn in regulating Cdk5 indirectly, rather than through direct activation [30].

Another debated site is Ser159, located within a region analogous to the activating T-loop of canonical Cdk family. Early biochemical studies have suggested this modification—mediated by a kinase distinct from the canonical Cdk-activating kinase (CAK), which is responsible for activation of canonical Cdk family—has been suggested to enhance Cdk5 activity, further highlighting its atypical nature [31]. However, structural analyses have shown that a phosphomimetic mutation (S159E) impairs p35 binding, leading to the alternative hypothesis that this phosphorylation may serve as a negative feedback mechanism to promote activator dissociation [32]. The precise physiological roles of these phosphorylation events remain an open question for future research.

### 2.4. Negative Regulators of Cdk5 Activity

Balancing these positive inputs, specific inhibitory pathways constrain Cdk5 signaling. For example, Cyclin E, a core cell cycle protein, can prevent Cdk5 from binding to p35 or p39 in post-mitotic neurons, thereby suppressing its kinase activity [33]. In addition, Glutathione S-transferase P1 (GSTP1) inhibits Cdk5 through two mechanisms: by directly competing with p35/p25 for binding, and by reducing the oxidative stress that can otherwise promote Cdk5 activity [34]. Furthermore, a Src- and protein kinase C delta (PKCδ)-dependent cascade can negatively regulate Cdk5. PKCδ can phosphorylate Cdk5 at Thr77 in response to upstream cues, leading to dissociation of Cdk5 from p35 and thereby inhibition of its activity [35].

**Figure 2:**
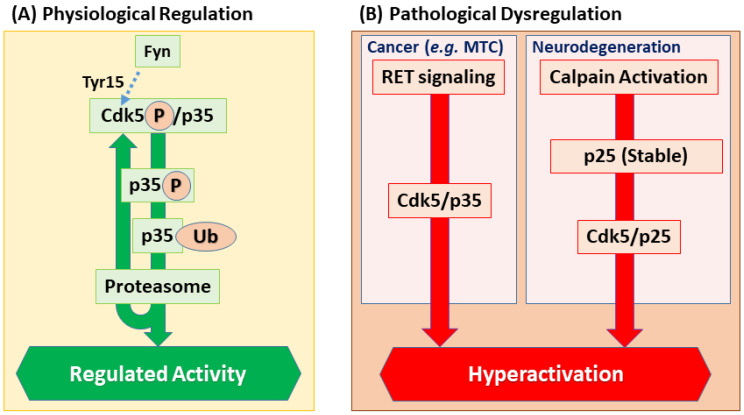
Upstream regulation of Cdk5 activity in physiological and pathological states. (**A**) In physiological settings, Cdk5 activity is transient and tightly controlled. Its activity is modulated by phosphorylation (e.g., at Tyr15 by Fyn, though this remains debated) and terminated via a negative feedback loop where Cdk5 phosphorylates p35, leading to its proteasomal degradation. (**B**) In pathology, this regulation is bypassed, leading to chronic hyperactivation. In cancer, oncogenic signaling can drive p35 overexpression. In neurodegeneration, neurotoxic insults trigger calpain-mediated cleavage of p35 into the hyperstable p25 fragment. Both pathways result in sustained, aberrant Cdk5 activity.

Collectively, the networks of transcriptional programs, stability control, post-translational modifications, and inhibitory mechanisms ensure that Cdk5 activity is finely tuned. In neurodevelopment, these regulatory networks generate tightly controlled, transient kinase activity for proper morphogenesis. In cancer, however, many of these same checkpoints are dysregulated, leading to chronic, aberrant Cdk5 activation that drives malignant traits (Figure 2) [22,23,24,29,30,31,32].

## 3. The Role of Cdk5 in Neurodevelopment

Building on these controls, we next examine how tightly choreographed Cdk5 activity shapes neurodevelopment. In the developing nervous system, Cdk5 is a key regulator that plays a critical role in nearly every step from the birth of a neuron to its integration into functional circuits. Its influence extends from controlling the cell cycle exit of neural progenitors to guiding neuronal migration, and finally to shaping the intricate morphology and connectivity of axons, dendrites, and synapses. This chapter will detail these multifaceted roles, which are achieved through the phosphorylation of a diverse array of substrates summarized in Table 1. We proceed from progenitor cell cycle exit (Section 3.1) to migration (Section 3.2) and to circuit assembly and plasticity (Section 3.3).

### 3.1. Controlling the Switch from Proliferation to Differentiation in Neural Progenitor Cells

The intricate architecture of the central nervous system, particularly the cerebral cortex, depends on the precise regulation of two opposing processes: the proliferation of neural progenitor cells and their subsequent differentiation into neurons. In the early stages of development, neural progenitors undergo self-renewal to expand the pool of cells that will form the brain. However, they must eventually make the critical transition from proliferation to differentiation to produce post-mitotic neurons, a process that Cdk5 helps to coordinate by regulating both cell cycle exit and neuronal differentiation.

In defined developmental contexts, Cdk5-dependent phosphorylation serves as a molecular switch on key signaling scaffolds: it directly phosphorylates Axin, a Wnt pathway scaffold, at Thr485 to regulate its nuclear accumulation and Axin–GSK3β interactions, biasing signaling toward neuronal differentiation and axon formation; and it phosphorylates Dixdc1, a partner of the schizophrenia-associated protein DISC1, at Ser250 to enhance Dixdc1–nuclear distribution element-like 1 (Ndel1) binding and promote neuronal migration, while leaving DISC1-dependent Wnt–GSK3β/β-catenin-driven progenitor proliferation largely unaffected [37,38,39]. Together, these data suggest a division of labor in early corticogenesis: Axin phosphorylation biases Wnt signaling toward neuronal differentiation and axon formation, whereas Dixdc1 phosphorylation primarily gates migration via Ndel1 binding without altering DISC1-dependent Wnt/β-catenin-driven progenitor proliferation.

Evidence for Cdk5’s role in promoting a post-mitotic state comes from Cdk5-null cortices, where neurons in the cortical plate aberrantly express cell cycle-associated markers such as Cyclin A, Cyclin D, Ki-67, and PCNA [65]. A key effector in this process is the Cdk family inhibitor p27kip1. Cdk5 contributes to the stabilization of the p27kip1 protein, which is critical for facilitating the transition from proliferation to differentiation and migration [36,66]. The importance of p27kip1 extends beyond simple cell cycle inhibition; it acts as a modular protein that independently promotes neuronal differentiation by stabilizing Neurogenin 2 and facilitates migration by inhibiting RhoA signaling [67]. By stabilizing p27kip1, Cdk5 therefore helps to coordinate these distinct but linked events. Collectively, Cdk5 tunes early neurodevelopment—from cell cycle exit to neuronal identity and migration—by modulating multiple signaling pathways.

### 3.2. Multistep Regulation of Neuronal Migration and Layer Formation

**Figure 3:**
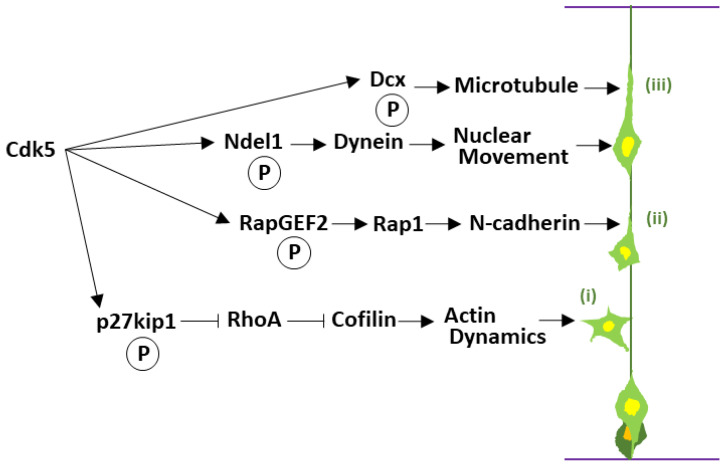
Cdk5 orchestrates distinct stages of neuronal migration. Cdk5 phosphorylates key substrates to control the multi-step migration process. (i) In multipolar cells, phosphorylation of p27kip1 regulates the RhoA-Cofilin pathway to control actin dynamics. (ii) During the bipolar transition, the RapGEF2 pathway modulates N-cadherin function. (iii) In locomoting neurons, phosphorylation of Ndel1 and Dcx governs nuclear movement and microtubule stability, respectively.

Having established a post-mitotic fate, neurons initiate a multistep migration program that Cdk5 coordinates at each step. The well-ordered six-layered structure of the mammalian cerebral cortex is the foundation of higher cognitive functions. This intricate architecture is formed during development as immature neurons, born in the ventricular zone lining the lateral ventricles deep within the developing brain, undertake a long-distance migration to their designated positions, arranging themselves into specific layers in an “inside-out” fashion according to their birth date [68]. This neuronal migration is a dynamic process involving a series of steps: after their final division, cells first adopt a “multipolar” morphology with multiple immature neurites [69], then undergo a transformation into bipolar “locomoting” neurons with a single thick leading process directed toward the pial surface [70], and migrate along radial glial fibers that act as guide rails [71]. Beginning with foundational studies on knockout mice, a large body of subsequent research, which will be discussed throughout this section, has firmly established Cdk5 as a central regulator at almost every stage of this complex migration process (Figure 3).

Systemic Cdk5 knockout mice exhibit perinatal lethality, and detailed analysis of their brains reveals a complete disruption of cerebral cortical lamination and a lack of cerebellar foliation [72,73]. This dramatic phenotype is primarily due to severe impairments in neuronal migration. In cerebral cortices deficient in either Cdk5 or its activator p35, the migration of the earliest-born neurons is relatively less affected. However, subsequently generated neurons fail to migrate past preexisting neurons and remain in deeper positions, resulting in a largely inverted “outside-in” layering [73,74]. Consistent with the essential role of Cdk5, the same phenotype is also observed in mice lacking both p35 and p39 [11].

Cdk5 precisely controls each step of migration by phosphorylating a wide array of downstream targets. In the first step, the formation of immature neurites in multipolar neurons, cells exhibit highly dynamic behavior, characterized by the continuous extension and retraction of multiple immature neurites as they probe their local environment. Cdk5 plays a critical role in regulating this process, functioning via p27kip1. The Cdk5-p27kip1 pathway suppresses the activity of the small GTPase RhoA, leading to the inactivation of its downstream Rho-kinase (ROCK). This results in the activation of the actin-depolymerizing factor Cofilin, which promotes the dynamic remodeling of actin filaments [36]. This precise control of the actin cytoskeleton enables the exploratory extension and retraction of immature neurites characteristic of multipolar cells (Figure 3, i).

In the second step, the morphological transition from a multipolar to a locomoting neuron, Cdk5 also plays a critical role. Cdk5 phosphorylates RapGEF2, which activates the small GTPase Rap1. Activated Rap1 is thought to promote the transport of the cell adhesion molecule N-cadherin to the cell surface [40]. This increased surface N-cadherin is critical for establishing the necessary adhesive connection to radial glial fibers, which serves as the track for the neuron’s subsequent long-distance migration [41] (Figure 3, ii). However, this regulation is exquisitely balanced; Cdk5 has also been reported to negatively regulate the strength of N-cadherin-mediated adhesion, likely via β-catenin phosphorylation [42]. This apparent paradox highlights Cdk5’s sophisticated, bidirectional control—promoting surface delivery while simultaneously modulating adhesion strength—a dynamic balance essential for cell migration.

In the third step, Cdk5 plays one of its most crucial functions: the regulation of long-distance migration of locomoting neurons (Figure 3, iii). The migration of locomoting neurons proceeds through a cycle of leading process extension and forward movement of the cell nucleus. Cdk5 has been shown to be necessary for the morphological changes specific to this mode of migration: the formation of a characteristic dilation (or swelling) at the proximal region of the leading process and the elongation of the nucleus and its subsequent movement into this dilation [75]. At the core of this regulation is the dynamic control of the cytoskeleton, particularly microtubules [76].

Cdk5 directly phosphorylates cytoskeletal regulators, including the lissencephaly-associated microtubule-binding protein Doublecortin (Dcx) and the dynein regulator Ndel1 [43,44,77]. Dcx, in particular, promotes microtubule polymerization and stability [78,79,80], but its phosphorylation at Ser297 by Cdk5 negatively regulates its binding affinity for microtubules [43]. This is critically important for preventing microtubules from becoming overly stabilized, thereby maintaining the flexible cytoskeletal dynamics required for cell movement [81]. Ndel1 is a regulator of the dynein complex [82], an intracellular motor protein that plays a central role in nuclear movement [83,84]. Cdk5 regulates the function of the dynein complex via Ndel1 phosphorylation, ensuring the smooth nuclear translocation of locomoting neurons [85]. This regulatory mechanism is so precise that even slight imbalances can cause severe defects. For example, Cdk5-mediated phosphorylation of nuclear distribution element 1 (Nde1), a paralog of Ndel1, is essential for proper neuronal lamination, and a schizophrenia-associated mutation in Nde1 has been shown to impair this specific phosphorylation, providing a direct molecular link between Cdk5 signaling and neurodevelopmental disorders [45]. In addition, phosphorylation of another key microtubule-associated protein, collapsin response mediator protein 2 (CRMP2), by Cdk5 is also critical for proper neuronal migration in both the cerebral cortex and hippocampus [46].

The significance of this precise neuronal positioning extends to the macroscopic morphogenesis of the brain; in gyrencephalic mammals like ferrets, Cdk5 function in upper-layer neurons is essential for cortical folding [86]. In summary, Cdk5 orchestrates neuronal migration, a process fundamental to brain morphogenesis, including macroscopic features like gyrification, by integrating control over the two major cytoskeletal systems of actin and microtubules, the motor proteins that transport intracellular cargo along them, and cell adhesion molecules.

### 3.3. Construction and Maturation of Neural Circuitry

**Figure 4:**
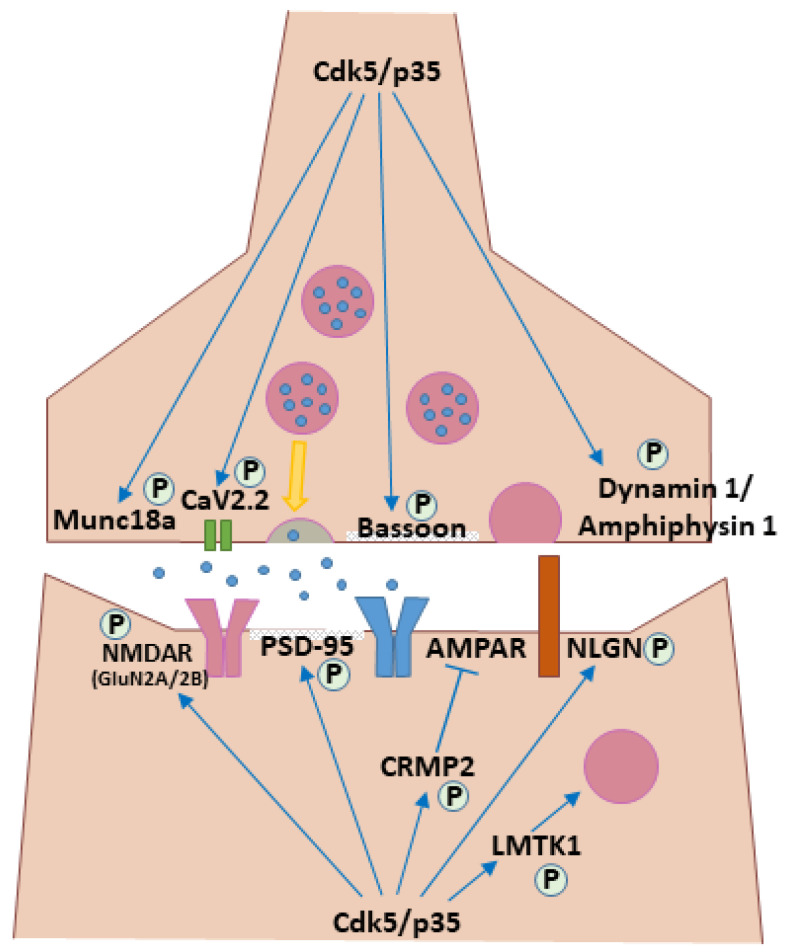
Cdk5 is a central hub regulating synaptic structure and function. Cdk5 phosphorylates a wide range of substrates at both presynaptic and postsynaptic sites to control synapse formation, maturation, and plasticity. Presynaptically (**top**), Cdk5 targets proteins involved in neurotransmitter release (e.g., Munc18a, CaV2.2) and vesicle recycling (e.g., Dynamin1). Postsynaptically (**bottom**), it regulates glutamate receptor trafficking (NMDAR, AMPAR), scaffolding (PSD-95), and synaptic adhesion (NLGN), thereby modulating synaptic strength and spine morphology. The images represent key synaptic components distinguished by color: the small blue dots represent neurotransmitters, and the pink circular structures represent membrane vesicles.

Once neurons reach their destinations, Cdk5’s role shifts from migration to neurite patterning, synapse formation and plasticity. Neurons specify an axon and initiate its outgrowth during the migratory phase; after reaching their final positions, they expand dendritic trees, further refine axonal projections, and form synapses to assemble functional circuits. Cdk5 plays a central role in both the physical wiring of neural circuits and the functional maturation of their synaptic connections, acting through a wide variety of substrates (Figure 4).

A foundational aspect of this role is its control over the establishment of neuronal polarity—the fundamental process by which a neuron specifies a single axon for transmitting signals and multiple, distinct dendrites for receiving them. Evidence for this critical function comes from mice with a cortex-specific deletion of Cdk5, where layer V pyramidal neurons fail to form a distinct apical dendrite, instead exhibiting an abnormal morphology with multiple shorter dendrites extending directly from the cell body [87]. This phenotype clearly indicates Cdk5’s essential role in establishing dendritic polarity, which in turn governs the development of characteristic dendritic patterns.

This fundamental role in morphogenesis is further reflected in axon guidance and outgrowth. Early studies using primary cultured cortical neurons showed that inhibiting Cdk5 activity suppresses neurite outgrowth, establishing its indispensability for this process [88]. This understanding has been reinforced in a human context using induced pluripotent stem cells (hiPSCs), where studies have shown that while Cdk5 deficiency does not impair neuronal differentiation, it paradoxically leads to an increase in neurite length. This reveals a more sophisticated role for Cdk5: not as a simple promoter of outgrowth, but as a critical regulator of morphogenesis that governs both extension and its limitation [89]. Indeed, this complex regulatory function is essential for proper circuit wiring in vivo. In the brains of Cdk5 knockout mice, the axonal projections of the thalamocortical pathway, a crucial connection between the thalamus and the cerebral cortex, are abnormal, and axons fail to reach their correct target areas [73]. This provides evidence that Cdk5 is involved not only in neurite extension at the single-cell level but also in axon guidance during brain-wide circuit formation. However, Cdk5 activity must also be negatively regulated. For instance, the RPM-1/FSN-1 ubiquitin ligase complex in *C. elegans* has been shown to inhibit Cdk5 to ensure proper axon termination, highlighting the importance of fine-tuning Cdk5 for correct neural wiring [90].

Cdk5’s involvement extends beyond the initial circuit wiring to the functional maturation of synapses and their subsequent plasticity. Cdk5 functions at both presynaptic and postsynaptic sites. At the presynaptic terminal, Cdk5 exerts a complex, bidirectional control over synaptic vesicle dynamics. On one hand, it can facilitate neurotransmitter release by directly phosphorylating the N-type Ca2+ channel CaV2.2 to increase Ca2+ influx and the number of docked vesicles [52]. On the other hand, Cdk5 also exerts several powerful braking mechanisms. It can attenuate release by phosphorylating P/Q-type voltage-dependent Ca2+ channels to reduce their activity [53]. It also regulates the endocytic phase of the synaptic vesicle cycle through phosphorylation of Dynamin1 and Amphiphysin1, a process thought to be critical for efficient vesicle recycling [48,49,50]. Furthermore, under conditions of high neuronal activity, Cdk5 acts as a homeostatic brake by suppressing the synthesis of the endosomal lipid PI(3)P, which is essential for presynaptic vesicle cycling and neurotransmission [91]. Beyond these opposing roles, Cdk5 also directly targets the core release machinery itself, phosphorylating Munc18a, a key component of the SNARE complex, and organizes the active zone via the scaffold protein Bassoon, underscoring its multifaceted control of the presynaptic terminal [47,51].

At the postsynaptic site, Cdk5 regulates the function and trafficking of NMDA-type glutamate receptors (NMDAR). These receptors are typically heterotetramers composed of two obligatory GluN1 subunits and two regulatory GluN2 subunits. The latter exist as different isoforms, such as GluN2A and GluN2B (also known as NR2A and NR2B, respectively), which confer distinct properties to the receptor. Cdk5 targets these GluN2 subunits through different mechanisms. For instance, a foundational study demonstrated that Cdk5 directly phosphorylates the GluN2A subunit at Ser1232, and that pharmacological inhibition of Cdk5 suppresses both NMDAR-mediated synaptic currents and the induction of long-term potentiation (LTP), directly linking Cdk5 activity to synaptic plasticity [54]. Cdk5 also regulates the surface expression of GluN2B-containing receptors, through multiple, distinct mechanisms. A key indirect pathway involves the interplay between the postsynaptic scaffold protein PSD-95 and the tyrosine kinase Src. By limiting the PSD-95–Src interaction, Cdk5 suppresses the phosphorylation of GluN2B at Tyr1472. Because this tyrosine phosphorylation normally inhibits the binding of the endocytic adaptor protein 2 (AP-2), Cdk5 permits AP-2 binding and facilitates the endocytosis of GluN2B-containing receptors, thereby keeping their surface levels low. Consequently, inhibition of Cdk5 blocks this endocytic pathway, leading to an accumulation of surface GluN2B [55]. Complementing this model, Cdk5 has also been shown to directly phosphorylate the GluN2B subunit at Ser1116. Disrupting this phosphorylation event, which is itself regulated by neuronal activity, similarly increases the surface levels of GluN2B and enhances memory formation, suggesting that Cdk5 utilizes multiple pathways to control the surface expression of NMDA receptors [56]. In addition to NMDAR, Cdk5 also precisely regulates the trafficking of AMPA receptors (AMPAR), another key glutamate receptor. Cdk5 phosphorylates CRMP2, which in turn inhibits CRMP2’s ability to deliver AMPAR to the postsynaptic surface, thereby negatively regulating synaptic efficacy [57].

Cdk5’s regulation of postsynaptic architecture is multifaceted. It targets not only the trafficking of receptors but also the core structural components, including the major scaffold protein PSD-95, to influence synaptic dynamics [58]. Its influence further extends to the synapse organizer machinery. It phosphorylates key postsynaptic adhesion molecules of the Neuroligin (NLGN) family, which bind to their presynaptic partners, Neurexins, to bridge the synaptic cleft and facilitate synapse formation and maintenance. Given their central role in circuit wiring, mutations in NLGNs, such as NLGN3 and NLGN4X, are strongly implicated in neurodevelopmental disorders. Cdk5-mediated phosphorylation of these NLGNs has profound functional consequences. For instance, phosphorylation of NLGN3 modulates its interaction with the postsynaptic signaling protein Kalirin-7 and its surface expression, ultimately affecting NLGN3-mediated synaptic currents in cultured neurons [59]. Similarly, phosphorylation of NLGN4X at Ser712 regulates dendritic spine morphology, specifically reducing the number of mature mushroom spines, and is associated with changes in miniature excitatory postsynaptic current (mEPSC) frequency [60]. Together, these findings demonstrate that Cdk5 fine-tunes synaptic structure and function by directly modifying core components of the synapse-organizing machinery. This link between Cdk5 dysfunction and neurodevelopmental deficits is further supported by findings that mice lacking the autism-associated gene Clcn4 exhibit synaptic deficits and reduced dendritic branching, which are accompanied by a notable decrease in Cdk5 expression, suggesting that disruption of the Cdk5 pathway may be a common feature in some forms of neurodevelopmental disorders [92]. Furthermore, Cdk5 regulates the supply of membrane components essential for spine morphogenesis. It achieves this by controlling the lemur tyrosine kinase 1 (LMTK1)-TBC1D9B-Rab11A cascade, a novel signaling pathway that modulates recycling endosome trafficking to the postsynaptic site [61].

Through these mechanisms, Cdk5 regulates forms of synaptic plasticity, such as long-term potentiation (LTP) and long-term depression (LTD), and thereby modulating higher brain functions including memory formation and extinction. This regulatory capacity is activated in response to external signals; for instance, acute exposure to corticotropin-releasing factor (CRF) utilizes the Cdk5 pathway to promote the rapid formation and stabilization of dendritic spines, linking Cdk5 to stress-related synaptic plasticity [93]. Strong NMDAR activation can trigger proteasome-dependent degradation of p35, transiently relieving Cdk5-mediated brakes and thereby facilitating LTP induction [94]. Thus, Cdk5 continues to regulate the formation, functional modulation, and activity-dependent remodeling of synapses long after the initial neural circuit is established. In doing so, it orchestrates the fundamental processes underlying circuit maturation and functional refinement. Crucially, this regulatory role is not confined to development but persists into adulthood, where Cdk5 is essential for maintaining the structural and functional integrity of mature neural circuits, as evidenced by its role in supporting motor control networks [95]. The precise regulation of Cdk5 is therefore crucial not only during development but also for the maintenance of the mature nervous system. Conversely, the deregulation of Cdk5 in the mature brain is a key contributor to neurodegenerative pathology. In Alzheimer’s disease models, for instance, hyperactivated Cdk5 excessively phosphorylates the protein Tau, leading directly to synaptic damage and cognitive decline, highlighting the critical importance of maintaining a fine balance of Cdk5 activity [64]. This deregulation is classically exemplified by the calpain-mediated proteolytic cleavage of its activator p35 into a smaller, more stable fragment called p25 (Figure 2B). This cleavage untethers the Cdk5 complex from the membrane, leading to its widespread mislocalization and hyperactivity throughout the cell. A similar cleavage product, p29, can be generated from p39, though its pathological role is less understood [96]. The conversion of p35 to p25, triggered by neurotoxic insults such as amyloid-β, leads to prolonged and aberrant Cdk5 hyperactivation, a key pathological driver in Alzheimer’s disease [14,97] (Figure 1B, ii). Crucially, the intricate molecular machinery that Cdk5 orchestrates for neuronal migration and circuit formation—governing the cytoskeleton, cell adhesion, and signaling cascades—provides the exact toolkit that is repurposed and distorted in the pathological context of cancer to drive invasion and metastasis, as we will explore in the next section. This role as a key regulator of neuronal homeostasis extends even further; for instance, Cdk5 also tunes circadian clocks by phosphorylating core components such as CLOCK (Thr451/Thr461) and PER2 (Ser394), thereby promoting their nuclear translocation and stabilizing clock output [62,63,98].

## 4. The Role of Cdk5 in Cancer

**Figure 5:**
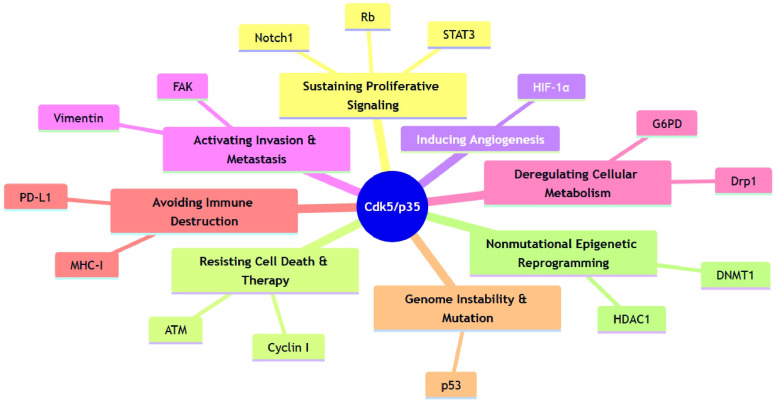
Cdk5 intersects with multiple hallmarks of cancer. Cdk5 and its activator p35 act as a core signaling node that promotes a wide array of oncogenic phenotypes, corresponding to the established and emerging hallmarks of cancer. This figure illustrates how Cdk5 drives each hallmark by phosphorylating representative substrates. For instance, it sustains proliferation by targeting Rb and STAT3, and promotes invasion by targeting FAK and Vimentin. A more comprehensive list of substrates is provided in Table 2.

In contrast to its constructive roles in neurodevelopment, Cdk5 is frequently repurposed in cancer, where it promotes a wide array of malignant phenotypes. To understand its multifaceted oncogenic functions, it is useful to place them within the conceptual framework of the “Hallmarks of Cancer”. Originally proposed by Hanahan and Weinberg, this framework outlines the core biological capabilities acquired by cells during tumorigenesis, such as sustained proliferation, invasion and metastasis, and evasion of cell death [99,100,101]. Remarkably, Cdk5 has emerged as a central signaling hub that contributes to nearly all of these established and emerging hallmarks. By phosphorylating a diverse set of substrates, Cdk5 drives cancer cell proliferation, enhances survival and therapeutic resistance, promotes invasion and metastasis, and actively shapes the tumor microenvironment. This chapter will detail these oncogenic roles, illustrating how Cdk5 acts as a key regulator of tumorigenesis by targeting the molecular machinery underlying each hallmark. Representative pathways are summarized in Figure 5 and detailed in Table 2.

### 4.1. Promotion of Cancer Cell Proliferation

In contrast to its role in promoting cell cycle exit in neural development, Cdk5 functions as a potent driver of proliferation in many cancers. This paradoxical functional switch is achieved by Cdk5’s direct intervention in cellular machinery controlling the cell cycle, signal transduction, and metabolism. Consistent with this pro-proliferative role, the expression of Cdk5 and its activator p35 is upregulated in a wide range of solid tumors—including breast, lung, prostate, pancreatic, melanoma, thyroid, and brain tumors. This elevated expression strongly correlates with cancer progression, lymph node metastasis, and poor prognosis; for instance, in bladder cancer, high Cdk5/p35 levels are associated with higher tumor grade and poor survival by promoting both proliferation and migration [132].

A primary mechanism by which Cdk5 drives proliferation is through the direct phosphorylation and deregulation of core cell cycle regulators. Its influence spans the entire cell cycle, from G1/S entry to mitotic completion. A remarkable example is its regulation of the retinoblastoma protein (Rb), a canonical tumor suppressor that acts as a gatekeeper of the G1/S transition. In the normal cell cycle, Rb is phosphorylated and inactivated by Cdk4/6 in response to pro-proliferative signals, thereby releasing E2F family transcription factors to drive cell cycle entry. In a striking instance of functional repurposing, Cdk5 can, at least in some contexts (e.g., MTC), functionally substitute for Cdk4/6 to phosphorylate Rb and promote proliferation [22]. This observation raises the possibility that tumors may escape inhibition by therapeutic Cdk4/6 inhibitors (e.g., palbociclib) via Cdk5-mediated Rb phosphorylation. Cdk5 further promotes cell cycle progression by inactivating Cdk family inhibitory proteins like p21^cip1^ in thyroid cancer [106] and facilitates DNA replication by phosphorylating the minichromosome maintenance protein 2 (MCM2), a key component of the DNA helicase complex, in pituitary tumors [105]. Consistent with a broad pro-proliferative role, Cdk5 knockdown in glioblastoma cells inhibits proliferation and induces apoptosis [133]. Its involvement extends even to the regulation of mitotic progression and fidelity; strikingly, and in contrast to its canonical activation mechanism, a recent report has described a novel Cdk5–cyclin B1 complex that appears to help ensure this process [21]. While the prevalence of this unconventional pairing remains to be elucidated, this finding is particularly notable as it raises the possibility that in the proliferative environment of cancer cells, Cdk5 can be repurposed to form functional complexes with classical cell cycle machinery.

In addition to targeting the core cell cycle machinery, Cdk5 amplifies pro-proliferative signals by activating key transcription factors and signaling pathways. A crucial target is signal transducer and activator of transcription 3 (STAT3), which Cdk5 directly phosphorylates at Ser727 to enhance its transcriptional activity in prostate cancer and MTC [102,103]. Cdk5 also activates other key oncogenic pathways, such as the Notch1 signaling pathway in pancreatic cancer [107]. Its influence extends to master oncogenes like c-Myc; in non-small cell lung cancer (NSCLC), for instance, Cdk5 has been reported to phosphorylate c-Myc at Ser62. This phosphorylation event prevents the binding of the tumor suppressor bridging integrator 1 (BIN1), thereby promoting Myc-dependent proliferation and neutralizing a key anti-tumor brake [109]. In prostate cancer, Cdk5 additionally targets the androgen receptor (AR), phosphorylating it at multiple sites to increase its stability and activity [103,104]. A synergistic interplay has also been observed where Cdk5-phosphorylated STAT3 further stabilizes AR, demonstrating Cdk5’s role as a mediator that integrates multiple pro-proliferative signals [103]. This mechanism is of particular clinical relevance in the context of therapeutic resistance. The PI3K/Akt pathway is a pro-survival signaling cascade that is frequently hyperactivated in cancer, making it a major therapeutic target. However, treatment with PI3K/Akt inhibitors can paradoxically activate Cdk5, leading to a compensatory enhancement of AR signaling that bypasses PI3K/Akt blockade and drives therapy resistance. This paradoxical activation occurs because Akt normally phosphorylates the Cdk5 activator p35, targeting it for degradation; PI3K/Akt inhibition therefore stabilizes p35, leading to increased Cdk5 activity [134].

Rapidly proliferating cancer cells must reprogram their metabolism to satisfy the high demand for energy and biosynthetic precursors. Cdk5 plays an essential role in supporting these metabolic adaptations by targeting key enzymes and signaling nodes.

For instance, Cdk5 directly modulates core metabolic pathways. In breast cancer, it phosphorylates G6PD, a rate-limiting enzyme in the pentose phosphate pathway, to maintain redox homeostasis [121]. Similarly, in glioblastoma, a key O-GlcNAc–Cdk5– acyl-CoA synthetase short-chain family member 2 (ACSS2) signaling pathway links acetate utilization to the lipid synthesis critical for tumor growth: O-GlcNAc transferase (OGT)-mediated O-GlcNAcylation enhances Cdk5 activity, which in turn phosphorylates ACSS2 at Ser267 to stabilize the enzyme and drive acetyl-CoA production [124]. This intersection of metabolism and survival is further exemplified in hematopoietic malignancies, where Cdk5-dependent phosphorylation of the BH3-only protein NOXA at Ser13 simultaneously restrains its pro-apoptotic function while shifting glucose flux toward the pentose phosphate pathway, a key anabolic and antioxidant pathway essential for cancer cell survival [114].

Beyond cytosolic metabolism, Cdk5 is also a critical regulator of mitochondrial dynamics and function. In hepatocellular carcinoma (HCC), for example, Cdk5 stabilization promotes metabolic reprogramming and cancer progression by modulating mitochondrial fission [122]. This is mediated, at least in part, through the phosphorylation of dynamin-related protein 1 (Drp1), a key GTPase that governs mitochondrial fission. Cdk5-dependent phosphorylation of Drp1 at a conserved Ser616-equivalent site (human S616; rodent S585) shifts mitochondrial dynamics toward fission—a change that can support metabolic reprogramming but, when excessive, trigger apoptosis, as discussed below (Section 6) [122,123,135].

The oncogenic potential of Cdk5 is thus multifaceted, contributing not only to these core metabolic programs but also to the emergence of tumor heterogeneity, as observed in pancreatic neuroendocrine tumors [136].

However, the role of Cdk5 in HCC is complex, underscoring its context-dependency. While most large-scale cohort studies report that increased Cdk5 expression is associated with tumor progression and poor clinical outcomes [110,115], recent immune studies have revealed a paradoxical, tumor-suppressive role where Cdk5 promotes the autophagic degradation of the immune checkpoint molecule programmed death-ligand 1 (PD-L1), thereby potentially enhancing anti-tumor immunity [126]. This highlights that the ultimate prognostic impact of Cdk5 in HCC is shaped by the interplay between the specific oncogenic pathways being engaged and the immune/microenvironmental context.

### 4.2. Promotion of Invasion and Metastasis

Beyond promoting cell proliferation, Cdk5 plays a direct role in the physical dissemination of cancer cells. Metastasis—the process by which cancer cells detach from the primary tumor and spread to other organs—is responsible for over 90% of cancer-related deaths [137,138,139]. Cdk5 can act at the earliest stages of this process by regulating the ‘migration–proliferation dichotomy,’ a critical decision point for cancer cells. For instance, upon epidermal growth factor (EGF) stimulation, Cdk5 phosphorylates Girdin, a key signaling scaffold, which simultaneously enhances pro-migratory Akt signals while dampening pro-proliferative mitogen-activated protein kinase (MAPK) signals, thereby biasing the cellular program toward invasion [140]. This illustrates how Cdk5 repurposes migration signaling modules. Indeed, both Girdin, a direct substrate of Cdk5 in this context, and the downstream Akt pathway are themselves critical regulators of distinct modes of neuronal migration. Girdin regulates the chain migration of neuroblasts along the rostral migratory stream (RMS), a pathway for newborn neurons to reach the olfactory bulb, while the Akt pathway is essential for the radial migration of cortical neurons [140,141,142].

Before cancer cell invasion, epithelial cells undergo an epithelial–mesenchymal transition (EMT), a process where epithelial cells acquire mesenchymal characteristics, enabling invasion and dissemination [143]. Although the requirement of EMT in cancer metastasis remains controversial [144], many studies have indicated that EMT is a critical step for cancer cell invasion and dissemination. Cdk5 has been identified as a key regulator of this process in breast cancer cells stimulated with transforming growth factor beta 1 (TGF-β1), a major inducer of EMT [116].

Once cancer cells acquire migratory potential, Cdk5 directly modulates cell adhesion and cytoskeletal dynamics. A key set of targets are components of focal adhesions, integrin-mediated cell-to-extracellular matrix adhesions required for cell migration. For instance, Cdk5 phosphorylates focal adhesion kinase (FAK) at Ser732—a phosphorylation event also critical for neuronal migration—and the adhesion protein talin at Ser425 [116,117,145]. The importance of Cdk5-mediated talin phosphorylation is evident in colorectal cancer, where it promotes cancer progression and metastasis [118]. The clinical relevance of the Cdk5–FAK pathway is highlighted by the finding that in KRAS-mutant lung cancer, acquired resistance to FAK inhibitors involves compensatory activation of the ERK5 pathway [146]. Beyond focal adhesions, Cdk5 also targets the cytoskeleton directly. In melanoma, for example, it phosphorylates the intermediate filament vimentin at Ser56, altering its structure to increase cell motility [119]. Furthermore, Cdk5 can amplify pro-invasive programs by activating downstream signaling cascades; in colorectal cancer, it directly phosphorylates ERK5 at Thr732 within its C-terminal transcriptional activation domain, a key step that enables ERK5-dependent activating protein 1 (AP-1) coactivation and subsequent upregulation of oncogenic targets including c-Myc, VEGFA, and MMP1 [108].

Collectively, by targeting these distinct cellular components—focal adhesions, the cytoskeleton, and signaling cascades—Cdk5 comprehensively regulates the molecular machinery required for cancer cell invasion and migration.

### 4.3. Cdk5 in the Tumor Microenvironment

In parallel with cell-intrinsic programs, Cdk5 also remodels the tumor microenvironment—blood vessels and immunity—that feeds back on tumor behavior. The progression of cancer is not solely dependent on the intrinsic properties of tumor cells but is profoundly influenced by a complex milieu known as the tumor microenvironment (TME). This environment, consisting of blood vessels, immune cells, fibroblasts, and extracellular matrix, maintains a dynamic crosstalk with cancer cells that can either suppress or promote malignancy. Emerging evidence reveals that Cdk5 is a key player in this interplay, acting not only within cancer cells but also on the surrounding stromal components to shape a pro-tumorigenic niche.

A critical function of Cdk5 in the TME is its direct role in promoting angiogenesis. Within endothelial cells, Cdk5 supports neovascularization by driving proliferation, survival, and Rac1-dependent migration [147,148]. Its expression in this context is itself regulated by the balance of microenvironmental signals, induced by pro-angiogenic factors like basic fibroblast growth factor (bFGF) from tumor cells and suppressed by endogenous inhibitors like angiostatin [147,149]. Furthermore, endothelial Cdk5 is critically involved in regulating vessel patterning by interfacing with the DLL4–Notch signaling pathway. Cdk5 is required for efficient Notch intracellular domain (NICD) generation and target-gene expression, adding a crucial layer of control over functional angiogenesis [150]. In parallel, Cdk5 acts within the cancer cells themselves to regulate a pro-angiogenic secretome. It achieves this by phosphorylating and stabilizing Hypoxia-inducible factor 1α (HIF-1α), a master transcription factor that potently induces the expression and secretion of VEGF, thereby recruiting new blood vessels to the tumor [115].

Beyond shaping the physical microenvironment, Cdk5 is a critical modulator of the interaction between cancer cells and the immune system. A striking example is observed in breast cancer brain metastasis, where astrocytes—a key component of the brain TME—upregulate Cdk5 in metastatic cancer cells. This, in turn, suppresses the expression of MHC-I molecules on the cancer cell surface, allowing them to become unrecognizable to and evade destruction by cytotoxic T cells and facilitating immune evasion and metastatic colonization of the brain [125].

Conversely, Cdk5 can also function to enhance anti-tumor immunity. In hepatocellular carcinoma, Cdk5 promotes the degradation of the immune checkpoint molecule PD-L1 through chaperone-mediated autophagy (CMA). By destabilizing PD-L1, Cdk5 prevents cancer cells from inactivating T cells, thus promoting immune surveillance [126]. However, this effect is highly context-dependent, as contradictory, pro-tumorigenic roles have been reported in other malignancies. In lung adenocarcinoma, Cdk5 appears to stabilize the PD-L1 protein [127], and in medulloblastoma, it promotes interferon-γ (IFN-γ)-induced PD-L1 transcription by suppressing its transcriptional repressors [151]. The reason for this divergence across different cancer types is not yet fully understood, but it may result from factors such as tissue-specific binding partners, differential crosstalk with other oncogenic pathways, the status of post-translational modifications that determine substrate specificity, and influences from the specific tumor microenvironment of each cancer type. This starkly opposing regulation of PD-L1 in different cancer types illustrates the profound complexity of Cdk5 signaling and underscores the need for context-specific investigation.

The role of Cdk5 in other key TME components, such as Cancer-Associated Fibroblasts (CAFs), remains less understood but represents a promising area for future research. Given Cdk5’s established role in regulating the cytoskeletal organization, cell adhesion, and motility, it is plausible that it could influence CAF activation, extracellular matrix deposition, or secretion of pro-tumorigenic factors that drive cancer progression [152]. Indeed, emerging evidence links Cdk5 to CAF-driven epigenetic programs. In breast cancer, CAF-derived TGF-β1 induces HOTAIR—a long non-coding RNA that recruits polycomb repressive complex 2 (PRC2)/ enhancer of zeste homolog 2 (EZH2) to deposit the repressive histone mark H3K27me3—which directs H3K27me3 to the CDK5RAP1 and early growth response protein 1 (EGR1) promoters, lowering their expression and associating with higher Cdk5 levels and pro-metastatic EMT [153]. Because CDK5RAP1, despite its name, primarily functions as a nuclear/mitochondrial tRNA 2-methylthio transferase, this HOTAIR/PRC2–Cdk5 link is likely indirect [154].

Beyond its impact on stromal cells like CAFs, Cdk5 also directly modulates immune cell function. In T-cells, for instance, Cdk5 can tune histone deacetylase (HDAC) activity to control IL-2 expression [155]. Furthermore, it regulates Foxp3, a transcription factor that controls the differentiation and survival of regulatory T-cell (Treg), through the phosphorylation of STAT3 at Ser727 [156].

Cdk5 also plays a key role in shaping the inflammatory milieu by regulating gene expression at the translational level. In myeloid cells, IFN-γ signaling activates Cdk5, which then orchestrates the stepwise phosphorylation of glutamyl-prolyl-tRNA synthetase (EPRS), a canonical component of the translation machinery. This phosphorylation triggers the release of EPRS from its primary complex and its assembly into the alternative GAIT (Gamma interferon-Activated Inhibitor of Translation) complex. The GAIT complex, in turn, selectively binds to and suppresses the translation of a battery of pro-inflammatory mRNAs, thereby providing a crucial checkpoint to temper tumor-promoting inflammation [157].

Collectively, these findings establish Cdk5 not merely as a cell-intrinsic oncogene, but as a crucial modulator of the tumor microenvironment. By controlling angiogenesis and immune surveillance, Cdk5 can impact the dynamics of the anti-tumor response, highlighting it as a significant, yet complex, therapeutic target. Beyond blood vessels, data also implicate Cdk5 in the development of lymphatic vessels, suggesting it may regulate additional routes for metastatic dissemination [158].

### 4.4. Therapeutic Resistance via DNA Damage Response Activation

Resistance to chemotherapy and radiotherapy is a major clinical challenge, and Cdk5 plays a central role in this process by modulating the DNA Damage Response (DDR) [159]. When cancer cells are exposed to DNA-damaging agents, Cdk5 kinase activity is significantly induced, often through upregulation of p35 expression [160]. Activated Cdk5 acts as a modulator for the DDR cascade. Its primary mechanism is the Cdk5-mediated direct phosphorylation of ATM kinase at Ser794, which enhances its autophosphorylation and full activation to initiate the downstream signaling cascade [110,111,161].

Cdk5’s role extends across multiple stages of the DDR. For example, it directly phosphorylates factors such as Replication Protein A 32 (RPA32) to protect single-stranded DNA [112]. In addition, Cdk5 is thought to be involved in transcriptional control of DNA repair machinery. By phosphorylating STAT3 at Ser727, Cdk5 enhances STAT3 transcriptional competence. Given that EGFR–STAT3 signaling upregulates the endonuclease Eme1 [162,163], Cdk5 may facilitate the inducible expression of DNA repair effectors in a STAT3-dependent and context-specific manner under genotoxic stress. This multifaceted roles of Cdk5 comprehensively support genomic integrity and facilitate cellular recovery from DNA damage.

Clinically, this Cdk5-mediated activation of the DDR leads to therapeutic resistance, establishing Cdk5 as a key factor in tumor cell survival under genotoxic stress. Cdk5’s contribution to therapeutic resistance, however, is not limited to the DDR pathways and can be mediated by alternative pathways. A notable example is found in cervical cancer, where resistance to the chemotherapeutic agent cisplatin is driven by the upregulation of Cyclin I. In this context, the Cyclin I-Cdk5 complex functions as a critical anti-apoptotic factor, protecting cancer cells from drug-induced cell death [113]. This finding highlights that a specific oncogenic output of Cdk5 is determined not only by the cellular context but also by its choice of regulatory partner, adding another layer of complexity to its role in cancer progression and therapeutic resistance.

Beyond its role in the DDR, Cdk5-mediated regulation of cytoskeletal dynamics has also been linked to resistance to microtubule-stabilizing agents like paclitaxel. Intriguingly, while paclitaxel treatment can itself promote an invasive phenotype, this effect can be attenuated by Cdk5 inhibition [164]. Consistently, Cdk5 knockdown has been shown to restore paclitaxel sensitivity in ovarian cancer models [165]. Furthermore, the expression levels of Tau, a Cdk5 substrate, have been proposed as a predictive biomarker for paclitaxel response in both breast and gastric cancers, with low Tau expression correlating with better outcomes [166,167].

### 4.5. Repurposing the Neurodevelopmental Toolkit in Cancer

A recurring theme in the oncogenic functions of Cdk5 is the repurposing of its physiological roles to drive malignant progression. The kinase utilizes the same cellular machinery from normal neurodevelopmental processes, but in the context of cancer, this leads to uncontrolled invasion and metastasis.

This duality is exemplified by Cdk5’s regulation of the cytoskeleton and cell adhesion. For instance, Cdk5-mediated phosphorylation of FAK at Ser732, an event required for neuronal migration [145], is repurposed in breast cancer to drive invasion [116]. Cdk5 also targets Talin by phosphorylating it at Ser425. This mechanism was shown to be required for the migration of SH-SY5Y neuroblastoma cells, providing a link between this phosphorylation event and the motility of cells of neuronal origin. The same molecular switch is repurposed in other malignancies to promote metastasis [117].

This repurposing extends beyond cell motility to transcriptional programs. The transcription factor STAT3 serves as an example of how Cdk5 can utilize signaling molecules with broad physiological relevance. While STAT3 is a critical regulator of cell fate and survival in diverse contexts, its physiological roles are exemplified in the nervous system. For instance, in the adult hippocampus, signaling via the ciliary neurotrophic factor (CNTF) and its downstream effector STAT3 is essential for maintaining the neural stem cell pool and promoting neurogenesis [168]. In this physiological setting, STAT3 activation is tightly controlled by cytokine availability. In contrast, its oncogenic potential is fully realized by hyperactive Cdk5 in some cancer types. Cdk5-mediated phosphorylation at Ser727 can lock STAT3 in a constitutively active state, transforming it from a regulated signal into a persistent engine for malignant proliferation [102,103].

This functional duality can exist even within the same cellular compartment. A striking example is observed in non-small cell lung cancer (NSCLC), where cytoplasmic Cdk5 can phosphorylate the tumor suppressor Deleted in Liver Cancer-1 (DLC1) on multiple serine residues (e.g., S120, S205, S422, S509). This enhances DLC1’s focal adhesion localization and its Rho GTPase-activating protein (Rho-GAP) activity, thereby exerting potent anti-oncogenic effects. This suggests that the overall oncogenic output of Cdk5 may depend on the availability of its substrates; in many tumors, its pro-tumorigenic role may become dominant precisely because tumor-suppressive substrates like DLC1 are downregulated [120].

These examples illustrate a fundamental principle: Cdk5 is a context-dependent tool. Its impact is defined by the cellular environment in which it operates. Understanding this repurposing of cellular machinery provides a strong conceptual framework for Cdk5’s dual roles and reinforces its significance as a therapeutic target in cancer.

## 5. Nuclear Functions of Cdk5

**Figure 6:**
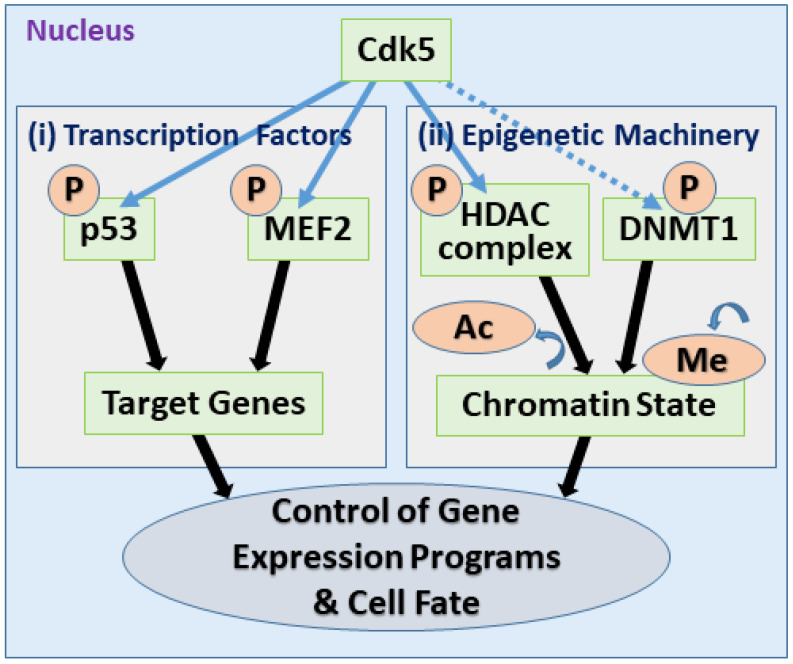
Nuclear Cdk5 regulates gene expression through dual mechanisms. Once in the nucleus, Cdk5 controls gene expression programs via two main pathways. (i) It directly phosphorylates transcription factors, such as p53 and MEF2, to modulate their activity. (ii) It targets the epigenetic machinery, including HDAC complexes and DNMT1, to alter chromatin state through histone acetylation (Ac) and DNA methylation (Me). The dotted arrow indicates a regulatory link requiring further validation.

While Cdk5 is well known for its cytoplasmic roles in regulating the cytoskeleton and membrane trafficking, multiple studies highlight the nucleus as a critical site of its activity. The subcellular localization of Cdk5 is a key determinant of its functional output, acting as a molecular switch that alters its substrate repertoire and biological consequences. This chapter explores the nuclear functions of Cdk5, focusing on its roles as a direct regulator of transcription factors and as a key modulator of the epigenetic machinery (Figure 6). These nuclear activities provide a mechanistic basis for its context-dependent duality, as best exemplified by the most striking illustration of this principle: its paradoxical function as a tumor suppressor in gastric cancer.

### 5.1. Direct Regulation of Transcription Factors

At the most direct level, nuclear Cdk5 modulates gene expression by phosphorylating various transcription factors, thereby controlling their activity, stability, or localization. As discussed in the context of cancer, Cdk5 phosphorylates STAT3 and the androgen receptor (AR), enhancing their pro-proliferative transcriptional programs [102,103]. However, its influence extends to other key transcription factors that govern cell fate decisions.

For example, Cdk5 can directly phosphorylate the tumor suppressor p53. A foundational study in a PC12 pheochromocytoma cell line, often used as a neuronal cell model, demonstrated that the hyperactive Cdk5/p25 complex phosphorylates p53, leading to an increase in both its protein levels and its transcriptional activity, thereby promoting the expression of pro-apoptotic genes like Bax [128]. This established a direct link between Cdk5 activity and the p53-mediated apoptotic pathway, although the precise outcomes may vary depending on the cellular context and other concurrent signals.

In the nervous system, Cdk5 exerts critical control over myocyte enhancer factor 2 (MEF2), a family of transcription factors essential for neuronal survival. Under pathological conditions, such as in models of Parkinson’s disease, hyperactivated Cdk5/p25 phosphorylates MEF2 at an inhibitory site, Ser444. This phosphorylation suppresses MEF2’s transcriptional activity, ultimately promoting dopaminergic neuron death. Crucially, expression of a Cdk5-unphosphorylatable mutant of MEF2 provides neuroprotection in vivo, establishing the Cdk5-MEF2 pathway as a key pathway in neurodegeneration [169]. These examples demonstrate that nuclear Cdk5 is positioned to directly translate upstream signals into specific transcriptional outcomes.

### 5.2. Control of the Epigenetic Machinery

Beyond direct transcription factor control, Cdk5 exerts broader, genome-wide control by targeting the core epigenetic machinery itself—the enzymes that write, erase, and read chemical marks on DNA and histones.

A key target is the epigenetic machinery controlling histone acetylation, particularly HDAC complexes, which generally lead to chromatin compaction and gene repression. Cdk5 exerts complex control over this system. Under pathological conditions driven by p25, hyperactive Cdk5 inhibits the activity of HDAC1, leading to aberrant cell cycle gene expression, DNA damage, and subsequent neuronal apoptosis [129]. One proposed mechanism for this regulation involves the phosphorylation of HDAC complex components; Cdk5 phosphorylates mSds3, an integral component of the HDAC complex, at Ser228. Suppression of Cdk5 activity attenuates aberrant histone acetylation and protects neurons from apoptosis, suggesting that Cdk5 fine-tunes the epigenetic states by modulating the function of HDAC complexes [130].

Furthermore, Cdk5 has been implicated in the regulation of DNA methyltransferase 1 (DNMT1), the primary maintenance methyltransferase responsible for faithful replication of existing DNA methylation patterns, in contrast to the de novo methyltransferases DNMT3A and DNMT3B, which establish new methylation patterns during development [170,171]. An in vitro study demonstrated that not only Cdk5, but also Cdk1 and Cdk2, can phosphorylate human DNMT1 at Ser154, a modification shown to be important for its enzymatic activity and protein stability. Consistent with a role for Cdk family kinases, pan-Cdk family inhibitor roscovitine (also known as seliciclib) was found to reduce endogenous DNMT1 phosphorylation in cells. While this suggests a potential link, the specific contribution of Cdk5 relative to other Cdk family in vivo remains to be elucidated. Nevertheless, given that aberrant DNA methylation is a hallmark of cancer, Cdk5 stands as a plausible candidate for the upstream regulator of this fundamental epigenetic process [131].

**Figure 7:**
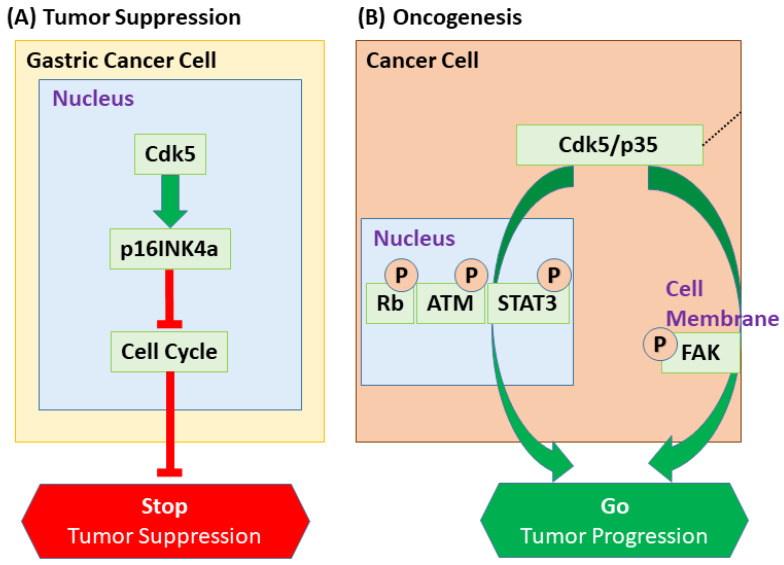
The functional duality of Cdk5 in cancer is determined by its subcellular localization. (**A**) In gastric cancer, nuclear Cdk5 acts as a tumor suppressor by inducing p16INK4a expression, which leads to cell cycle arrest and tumor growth inhibition. (**B**) In many other cancers, cytoplasmic and membrane-associated Cdk5/p35 functions as an oncogene. It drives proliferation, metastasis, and therapy resistance by phosphorylating a diverse range of substrates in different cellular compartments, including Rb and STAT3 in the nucleus and FAK at the cell membrane. Green arrows indicate activation or promotion pathways, while red T-bars indicate inhibition.

The most striking example of Cdk5’s nuclear function is its paradoxical role as a tumor suppressor in gastric cancer. In this malignancy, Cdk5 expression is reduced, and the protein is largely excluded from the nucleus, which correlates with poor prognosis [172,173]. Mechanistically, nuclear Cdk5 acts as a tumor suppressor by inducing the expression of the Cdk family inhibitor p16INK4a, thereby inhibiting the cell cycle [172] (Figure 7). This protective pathway is governed by a dynamic nucleocytoplasmic shuttling mechanism: Cdk5 lacks an intrinsic nuclear localization signal (NLS) and can enter the nucleus possibly via binding to p27, whereas two nuclear export signals (NESs) mediate CRM1-dependent export to promote cytoplasmic localization [172,174]. Consistent with this, a small molecule (NS-0011) that binds the NES region of Cdk5 promotes its nuclear accumulation and suppresses tumor growth, validating this transport machinery as a therapeutic target [172]. This tumor-suppressive function also extends to enhancing treatment efficacy, as nuclear Cdk5 has been shown to promote apoptosis and attenuate chemoresistance via the DP1–E2F1 axis [175].

In summary, the nuclear functions of Cdk5 reveal its role as a central regulator of gene expression programs. By directly targeting both transcription factors and the epigenetic enzymes that control the entire chromatin environment, nuclear Cdk5 can exert profound and lasting changes in cell fate, providing a deeper mechanistic understanding of its context-dependent duality.

## 6. Conclusions and Future Perspectives: The Duality of Cdk5 and Its Implications for Cancer Therapy

As described in this review, Cdk5 is a prime example of a multifunctional kinase whose role is determined by cellular context. In neural development, it orchestrates cell cycle arrest, differentiation, migration, and maturation. In cancer, these functions are repurposed to promote malignancy, driving proliferation, enhancing metastasis, supporting angiogenesis, and conferring resistance to therapy.

However, this functional switch is not arbitrary; it is governed by a sophisticated interplay of molecular determinants. A central theme emerging from this review is that the “duality” of Cdk5 is dictated by at least three key factors: its subcellular localization, its choice of activating partner, and the landscape of available substrates. The most striking illustration of this principle is the paradoxical role of Cdk5 in gastric cancer, where its nuclear localization drives a tumor-suppressive program by inducing p16INK4a expression. This stands in contrast to its predominantly cytoplasmic, pro-tumorigenic functions in other malignancies, where it phosphorylates substrates involved in motility and proliferation (Figure 7). Similarly, the identity of its activator can redefine its function; while the p35/Cdk5 complex is the canonical driver in both neurodevelopment and many cancers, association with noncanonical partners like Cyclin I can confer specific functions such as chemoresistance. Finally, post-translational modifications that ensure transient activity in neurons are often dysregulated in pathology, leading to sustained, aberrant signaling. Collectively, these mechanisms indicate that Cdk5’s functional output is context-dependent, being shaped by its subcellular localization, activator identity, post-translational state, and substrate availability within the cellular environment.

Furthermore, exploring how Cdk5 integrates with other signaling pathways known for their dual roles in cancer, such as the TGF-β pathway, represents an important avenue for future research. Given that Cdk5 phosphorylates p53 and is involved in TGF-β-induced EMT, investigating whether Cdk5 modulates the functional switch of TGF-β signaling through the interplay within the SMAD-p53/p63 complex could reveal deeper mechanisms of oncogenic reprogramming.

As highlighted in this review, the multifaceted oncogenic roles of Cdk5 have established it as a promising, albeit complex, therapeutic target. The potential of Cdk5 inhibition has been demonstrated in numerous preclinical studies, initially using broad-spectrum Cdk family inhibitors like roscovitine/seliciclib, which was shown to sensitize non-small cell lung cancer cells to radiation [176] and suppress tumor growth in hepatocellular carcinoma when combined with chemotherapy [110].

More recent multi-Cdk family inhibitors have also shown promise. Dinaciclib, for example, enhances cisplatin efficacy in testicular cancer [177] and suppresses pancreatic tumor growth by inhibiting the β-catenin/YAP axis [178]. Other agents, such as AT7519 and the Cdk2/5 inhibitor CP668863 (20-223), have demonstrated efficacy in preclinical models of neuroblastoma and colorectal cancer, respectively [179,180]. However, a critical limitation of these multi-targeted agents is their lack of specificity, which complicates the attribution of therapeutic effects to Cdk5 inhibition alone and raises concerns about off-target toxicities. This highlights the urgent need for more selective therapeutic strategies.

One promising avenue lies in targeting the specific protein–protein interactions required for Cdk5 activation. Peptide inhibitors such as CIP/p5/TFP5, designed to selectively disrupt Cdk5–p25/p35 interactions, have already shown in vivo efficacy in neurodegeneration models [181,182], highlighting a path toward more target-selective modalities. Notably, TFP5 shows in vivo preference for Cdk5/p25 over Cdk5/p35, likely because the p35 N-terminal p10 region binds Munc18 and sequesters the peptide, thereby sparing the physiological complex [183]. This strategy has also shown promise in oncology; in glioblastoma models, the related p35-derived peptide TP5 attenuates Cdk5/p25 hyperactivity, reduces tumor cell viability (partly by inhibiting ATM signaling), and demonstrates powerful synergy with standard-of-care agents like temozolomide and radiation [184].

Looking forward, therapeutic development is shifting toward more precise strategies. Proteolysis-Targeting Chimeras (PROTACs), for instance, represent a novel approach to selectively degrade, rather than just inhibit, Cdk5 [185,186]. In principle, PROTACs could be designed to preferentially target oncogenic cytoplasmic Cdk5 pools while sparing nuclear tumor-suppressive Cdk5. A complementary strategy for cancers like gastric cancer, where nuclear Cdk5 is beneficial, would be to use small molecules that modulate its nucleocytoplasmic shuttling to enforce its tumor-suppressive function [172].

These innovative approaches, combined with strategies to overcome resistance to existing therapies like Cdk4/6 inhibitors [22] or to synergize with immunotherapy, pave the way for a new generation of context-specific Cdk5-targeted treatments. However, the dual nature of Cdk5 necessitates a cautious approach, prioritizing the development of peripherally restricted or BBB-impermeant inhibitors to mitigate potential neurological side effects when targeting cancers outside the central nervous system. Successfully translating Cdk5 inhibition into a viable clinical strategy will require a deep, context-dependent understanding of its pleiotropic functions.

In summary, research on Cdk5 offers significant insights for both basic science and clinical oncology. Further elucidation of the molecular basis of its functional duality will be critical for developing novel, context-specific therapeutic strategies that target Cdk5.

## Figures and Tables

**Table 1:** Key Mediators of Cdk5 Signaling in Neuronal Development and Function.

Biological Process	Molecule	Biological Role	Ref.
**Neural Progenitor Regulation**			
Cell Cycle Exit and Differentiation	p27kip1 (CDK Inhibitor)	Phosphorylation at Ser10 stabilizes the protein to regulate actin dynamics for neuronal migration.	[36]
	Axin (Wnt Signaling)	Phosphorylation at Thr485 biases Wnt signaling from proliferation toward differentiation and axon formation.	[37,38]
	Dixdc1 (DISC1 Partner; Scaffold)	Phosphorylation at Ser250 enhances Ndel1 binding to promote migration, not affecting DISC1–Wnt/proliferation.	[39]
**Neuronal Migration**			
Multipolar to Bipolar Transition	RapGEF2 (Guanine Nucleotide Exchange Factor)	Activates Rap1, initiating the process of N-cadherin surface trafficking.	[40]
Locomotion and Cytoskeletal Control	N-cadherin (Adhesion Molecule)	Establishes and maintains adhesion to radial glia, providing the physical track for sustained locomotion.	[41]
	β-catenin (Adhesion Complex)	Phosphorylation negatively regulates N-cadherin-mediated cell adhesion.	[42]
	Doublecortin (Dcx) (Microtubule Regulator)	Phosphorylation at Ser297 modulates microtubule binding to ensure cytoskeletal flexibility during movement.	[43]
	Ndel1 and Nde1 (Dynein Regulators)	Control the dynein motor complex to drive nuclear translocation (nucleokinesis).	[44,45]
	CRMP2 (Microtubule Regulator)	Phosphorylation at Ser522 contributes to microtubule assembly and proper neuronal migration.	[46]
**Synapse Maturation, Plasticity, and Homeostasis**			
Presynaptic Function	Munc18a (SNARE Regulator)	Modulates the SNARE complex to regulate neurotransmitter release.	[47]
	Dynamin 1 (Endocytosis)	Phosphorylation at Ser774/Ser778 regulates synaptic vesicle endocytosis.	[48,49]
	Amphiphysin 1 (Endocytosis)	Phosphorylation regulates clathrin-mediated endocytosis for synaptic vesicle recycling.	[49,50]
	Bassoon (Scaffold)	Organizes the presynaptic active zone and coordinates Cdk5 and PKA signaling.	[51]
	CaV2.2 (N-type Ca^2+^ Channel)	Phosphorylation increases Ca^2+^ influx and neurotransmitter release.	[52]
	P/Q-type Ca^2+^ Channels	Phosphorylation reduces channel activity, dampening neurotransmitter release.	[53]
Postsynaptic Function	GluN2A (NMDAR Subunit)	Phosphorylation at Ser1232 directly enhances receptor function, contributing to long-term potentiation (LTP).	[54]
	GluN2B (NMDAR Subunit)	Promotes endocytosis and reduces surface levels via direct (Ser1116) and indirect (PSD-95/Src) mechanisms.	[55,56]
	CRMP2 (Microtubule Regulator)	Phosphorylation at Ser522 negatively regulates the surface delivery of AMPA receptors.	[57]
	PSD-95 (Scaffold)	Phosphorylation regulates scaffold dynamics and synaptic structure.	[58]
	Neuroligins (NLGN3, NLGN4X) (Synapse Organizers)	Phosphorylation modulates protein interactions, surface expression, and spine morphology.	[59,60]
	LMTK1 (Kinase)	Controls endosome trafficking to supply membrane for dendritic spine growth.	[61]
Circadian Rhythm Regulation	CLOCK (Transcription Factor)	Phosphorylation at Thr451/Thr461 promotes nuclear translocation and transcriptional activity.	[62]
	PER2 (Transcription Factor)	Phosphorylation at Ser394 facilitates nuclear entry and stabilizes the clock.	[63]
**Pathological Dysregulation**			
Pathological Activation	p25 (Activator Fragment)	Forms a hyperactive and mislocalized complex with Cdk5, driving neurotoxicity.	[14]
Pathological Substrate	Tau (Microtubule Regulator)	Aberrant hyperphosphorylation by Cdk5/p25, leading to synaptic damage.	[64]

**Table 2:** Oncogenic Functions of Cdk5 Mapped to Hallmarks of Cancer.

Hallmark of Cancer	Molecule	Mechanism of Action	Cancer Type	Ref.
**Sustaining Proliferative Signaling**	Rb (Tumor Suppressor)	Phosphorylation inactivates Rb to promote G1/S transition, substituting for Cdk4/6 in some contexts.	Medullary Thyroid	[22]
	STAT3 (Transcription Factor)	Phosphorylation at Ser727 activates transcription and proliferation.	Prostate, Medullary Thyroid	[102,103]
	Androgen Receptor (AR) (Nuclear Receptor)	Phosphorylation increases protein stability and transcriptional activity.	Prostate	[103,104]
	MCM2 (DNA Replication Factor)	Phosphorylation promotes DNA replication.	Pituitary	[105]
	p21cip1 (CDK Inhibitor)	Phosphorylation inactivates p21cip1 to promote cell cycle progression.	Thyroid	[106]
	Notch1 (Signaling Receptor)	Phosphorylation positively regulates Notch1 signaling pathway activity.	Pancreatic	[107]
	ERK5 (Kinase)	Phosphorylation at Thr732 enables AP-1 coactivation and c-Myc upregulation.	Colorectal	[108]
	c-Myc (Protooncogene)	Phosphorylation at Ser62 prevents the tumor suppressor BIN1 binding, disinhibiting proliferation.	NSCLC	[109]
**Resisting Cell Death & Therapy**	ATM (DDR Kinase)	Phosphorylation at Ser794 directly activates ATM to initiate the DNA Damage Response (DDR).	Multiple	[110,111]
	RPA32 (ssDNA-Binding Protein)	Phosphorylation protects single-stranded DNA (ssDNA) during genotoxic stress.	Multiple	[112]
	Cyclin I (CDK-binding)	A non-canonical anti-apoptotic complex with Cdk5 confers cisplatin resistance.	Cervical	[113]
	NOXA (BH3-only Protein)	Phosphorylation at Ser13 restrains apoptosis while shifting metabolism to the pentose phosphate pathway.	Hematopoietic	[114]
**Inducing Angiogenesis**	HIF-1α (Transcription Factor)	Phosphorylation stabilizes the protein, leading to upregulation of VEGF.	Multiple	[115]
**Activating Invasion & Metastasis**	FAK (Integrin-binding)	Phosphorylation at Ser732 regulates focal adhesions and EMT.	Breast	[116]
	Talin (Integrin-binding)	Phosphorylation at Ser425 modulates integrin activation and cell migration.	Colorectal	[117,118]
	Vimentin (Cytoskeleton)	Phosphorylation at Ser56 remodels intermediate filaments to increase cell motility.	Melanoma	[119]
	DLC1 (Tumor Suppressor)	Phosphorylation enhances its Rho-GAP activity, exerting context-dependent anti-oncogenic effects.	NSCLC	[120]
**Deregulating Cellular Metabolism**	G6PD (Metabolic Enzyme)	Phosphorylation activates the pentose phosphate pathway for redox balance.	Breast	[121]
	Drp1 (Mitochondrial Fission)	Phosphorylation at the conserved Ser616-equivalent site drives mitochondrial fission and creates a therapeutic vulnerability.	Colorectal, Hepatocellular	[122,123]
	ACSS2 (Metabolic Enzyme)	Phosphorylation at Ser267 stabilizes the enzyme, driving acetyl-CoA production from acetate.	Glioblastoma	[124]
**Avoiding Immune Destruction**	MHC-I (Antigen Presentation)	Astrocyte-induced downregulation of surface MHC-I enables T-cell evasion.	Breast (Brain Metastasis)	[125]
	PD-L1 (Immunosuppression)	Context-dependent regulation: promotes degradation (Hepatocellular) or stabilization (Lung).	Hepatocellular, Lung	[126,127]
**Genome Instability & Mutation**	p53 (Tumor Suppressor)	Dual regulation: pro-apoptotic in some contexts; may contribute to DDR dysregulation in others.	(General)	[128]
**Nonmutational Epigenetic Reprogramming**	HDAC1 (Epigenetic Modifier)	Inhibition of deacetylase activity (in pathological contexts) leads to aberrant gene expression and DNA damage.	(General)	[129]
	mSds3 (HDAC Complex Component)	Phosphorylation at Ser228 modulates HDAC complex and histone acetylation.	(General)	[130]
	DNMT1 (Epigenetic Modifier)	Phosphorylation at Ser154 shown in vitro; in vivo oncogenic relevance requires validation.	(General)	[131]

## Data Availability

No new data were created or analyzed in this study.

## Abbreviations

The following abbreviations are used in this manuscript:

ACSS2	Acyl-CoA synthetase short-chain family member 2
AMPAR	AMPA receptor
AP-1	Activating protein 1
AP-2	Adaptor protein 2
AR	Androgen receptor
ATM	Ataxia telangiectasia mutated
BBB	Blood–brain barrier
bFGF	basic fibroblast growth factor
BIN1	Bridging integrator 1
CAFs	Cancer-associated fibroblasts
CAK	Cdk-activating kinase
CCNI	Cyclin I
CCNI2	Cyclin I-like
Cdk	Cyclin-dependent kinase
CMA	Chaperone-mediated autophagy
CNTF	Ciliary neurotrophic factor
CRF	Corticotropin-releasing factor
CRM1	Chromosome region maintenance 1
CRMP2	Collapsin response mediator protein 2
Dcx	Doublecortin
DDR	DNA damage response
DLC1	Deleted in liver cancer 1
DNMT1	DNA methyltransferase 1
Drp1	Dynamin-related protein 1
EGF	Epidermal growth factor
EGR1	Early growth response protein 1
EMT	Epithelial–mesenchymal transition
EPRS	Glutamyl-prolyl-tRNA synthetase
ERK5	Extracellular signal-regulated kinase 5
EZH2	Enhancer of zeste homolog 2
FAK	Focal adhesion kinase
GAIT	Gamma interferon-activated inhibitor of translation
GSTP1	Glutathione S-transferase P1
HCC	Hepatocellular carcinoma
HDAC	Histone deacetylase
HIF-1α	Hypoxia-inducible factor 1α
hiPSC	human induced pluripotent stem cell
IFN-γ	Interferon-γ
LMTK1	Lemur tyrosine kinase 1
LTP/LTD	Long-term potentiation/depression
MAPK	Mitogen-activated protein kinase
MCM2	Minichromosome maintenance protein 2
MEF2	Myocyte enhancer factor 2
mEPSC	miniature excitatory postsynaptic current
MHC-I	Major histocompatibility complex class I
MTC	Medullary thyroid carcinoma
Nde1	Nuclear distribution element 1
Ndel1	Nuclear distribution element-like 1
NES	Nuclear export signal
NICD	Notch intracellular domain
NLGN	Neuroligin
NLS	Nuclear localization signal
NMDAR	NMDA receptor
NSCLC	Non-small cell lung cancer
OGT	O-GlcNAc transferase
PD-L1	Programmed death-ligand 1
PI(3)P	Phosphatidylinositol 3-phosphate
PKA	Protein kinase A
PKCδ	Protein kinase C delta
PRC2	Polycomb repressive complex 2
PROTAC	Proteolysis-targeting chimera
PSD-95	Postsynaptic density protein-95
Rb	Retinoblastoma protein
Rho-GAP	Rho GTPase-activating protein
RMS	Rostral migratory stream
RPA	Replication protein A
SNARE	soluble N-ethylmaleimide-sensitive factor attachment protein receptor
ssDNA	single-stranded DNA
STAT3	Signal transducer and activator of transcription 3
TGF-β1	Transforming growth factor β1
TME	Tumor microenvironment
Treg	regulatory T cell
YAP	Yes-associated protein
